# Identification of Olfactory Nuisance of Floor Products Containing Bitumens with the TD–GC–MS/O Method

**DOI:** 10.3390/ma15030959

**Published:** 2022-01-26

**Authors:** Mateusz Kozicki

**Affiliations:** Building Research Institute, Filtrowa 1 Street, 00-611 Warsaw, Poland; m.kozicki@itb.pl; Tel.: +22-57-96-187

**Keywords:** olfactometry, bitumens, odourant, TD–GC–MS, emission, IAQ

## Abstract

The adopted TD–GC–MS/O method helps determine the correlation between the odour signals and compounds separated on the chromatographic column, from the analysed gas mixture. It is possible to compare the retention times at which the odour signals were identified with the retention time of eluting compounds, when the test system and matrix are known. The presented study describes the details of representative samples obtained from (1) indoor air samples from a room where floor materials containing bitumen are present, (2) wooden floor staves placed in an emission chamber, and (3) fragments (chips) of the materials mentioned above, placed in glass tubes, exposed to an elevated desorption temperature. The results, presented in the paper, describe the identified odours and their intensity and assign chemical compounds to each odour, indicating their likely source of origin. The results presented in the manuscript are intended to show what methodology can be adopted to obtain intense odours from the tested samples, without losing the sensitivity derived from GC–MS. The manuscript presents representative results—case studies. The results for various types of samples were not very reproducible, related to the complex matrix of bituminous products. The enormity of compounds present in tar adhesives makes it possible to indicate only the groups of compounds that emit from these systems. They include, primarily, aliphatic, aromatic and heteroaromatic hydrocarbons, particularly Naphthalene and Phenol derivatives.

## 1. Introduction

Establishing unambiguous criteria for olfactory nuisance is hugely challenging. Construction products and interior design objects, which emit volatile organic compounds (VOC), are the most common causes of indoor air pollution in buildings [1,2,3,4]. The indoor air quality influences the inhabitants′ health and comfort. Studies by Wargocki et al. [5,6] and Shaughnessy et al. [7], show that poor air quality has a negative impact on office staff′s performance. This is why a growing demand is observed for technical equipment to measure, maintain and control indoor air quality. VOCs are responsible for the odour sensed by the users, on the condition that their concentration exceeds the odour detection threshold [8]. The intensity of an odour mixture, such as an air sample, can be determined based on the intensity of individual odours in the mixture. This way, at concentrations exceeding the detection threshold, the mixture′s odour intensity is lower than the total odour intensity of individual ingredients. The phenomenon is called hyperaddition or synergism [9].

The paper presents the results of air quality tests in a renovated office room, for the presence of compounds formed in wooden structures impregnated with tar compositions and bituminous sealants, containing hydrocarbon-based solvents. In order to confirm the source of VOC emission, the material samples were collected in the same rooms where air measurements were performed and tested in emission chambers. The most volatile polycyclic aromatic hydrocarbon, naphthalene, is a popular compound related to indoor air contamination, with the abovementioned materials [10]. A growing number of papers describe the harmfulness and effects of inhalation exposure to naphthalene and its derivatives [11,12,13]. Long-term exposure may occur among smokers and non-smokers exposed to tobacco in their environment, as well as among people working in areas where high concentrations of naphthalene are present (production of mothballs or creosote impregnating facilities) [14].

There are many methods of air sampling for the testing and identification of compounds in the collected samples, which help identify a potential source of unpleasant odour and understand the problem, once combined with other information, concerning the sampling location. The methodology of the likelihood of determination was established as part of the standardisation of the olfactometric measurement procedures. It is described by EN 13725:2003 [15]. However, the procedure focuses mainly on the air sampling methods in open spaces and is principally used for field tests, in response to people′s complaints about odours from agriculture, production, services, catering etc. According to the data provided by the Central Statistical Office [16], animal breeding and rearing is the agricultural domain with the highest odour nuisance. The following areas were identified as problematic olfactorily: meat and animal feed production plants—18% of complaints; poultry and swine breeding—12% of complaints and municipal waste and sewage—16% of complaints [16]. The standard [15] specifies exact requirements for persons who assess the odour (selection procedure), the minimum number of people in the assessing teams, the number of repetitions required and other conditions for odour detection threshold tests (individual and team values). A team is a group of at least four people with a similar sense of smell. Odour assessors who fulfil specific selection criteria are accepted into the teams. Therefore, stating, according to the method and conditions set out in the standard, that P = 0.5, means that one European Odour Unit (ouE) is present in one cubic metre. The likelihood of detecting the odour of air containing any pollutants is then the same as the likelihood of detecting the odour of air containing 123 µg of n-butanol (reference odourant).

Dynamic olfactometry is divided into direct (field) olfactometry, where measurements are carried out in real time (field measurements), with no delay. The stream of fragrant gas reaches the diluting apparatus and is diluted with a stream of neutral gas. This helps avoid errors related to sampling in analysis bags and changes in the sampled mixture’s composition during transport. Indirect (laboratory) olfactometry is the other type, where gas is sampled into foil bags and transported for analysis; this method is only used for high concentrations because odourants vary in time too much for low concentrations. In addition, transporting samples long distances poses some problems because of the chemical reactions occurring in the analysis bags [15,17].

Generally, the measurements are divided into short- and long-term ones (based on diffusion samples). Active sampling is among the short-term air sampling methods involving aspiration of known air volumes with aspirators (suction apparatuses) onto sorption tubes, with standardised dimensions. A GC–MS system, coupled with a thermal desorption (TD) system, is used to determine volatile organic compound content in the samples. In addition, the so-called cold-trap system, where compounds are concentrated by freezing, helps determine low concentrations.

The ISO 16000 series standards apply to different aspects of indoor air testing. Currently, the series contains 44 parts. The odour tests on construction materials are covered by ISO 16000-28: 2012 [18]. The referenced standard specifies the requirements for collecting air samples for odour assessment described in ISO 16000-9: 2006 [19]. The standard [18] uses two odour acceptability aspects, which determine the odour nuisance or acceptance on a −1 to +1 scale, and the perceived odour intensity that determines the odour intensity, regardless of its type and nuisance, compared to the odour intensity of air mixture with reference substances, such as acetone or n-butanol on a *pi* scale. A hedonic tone is an alternative to determine if the odour is perceived as pleasant or unpleasant on a nine-point perception scale, from −4 to +4. The standard [18] assumes that the odour assessment interface consists of a diffuser connected to the chamber outlet but also accepts other interfaces, such as odour masks, used in situations where the airflow rate in the test chambers does not fulfil the requirements for the airflow rate from the diffuser, e.g., for large construction products. Diffusers and masks must be airtight, made of odourless materials, such as stainless steel or glass, non-adsorptive (must not adsorb compounds on their surface), and the interface must not have its own emission that could come into contact with the tested air sample (non-permeable). Sample containers intended for collecting and transferring the samples from the test chamber to the place of their detection by panellists have to fulfil similar requirements. The materials recommended for transferring the samples include the tetrafluoroethylene hexafluoropropylene copolymer (FEP), polyvinyl fluoride (PVF) and polyethylene terephthalate (PET). It is assumed that the assessment should be made as quickly as possible after collecting the samples (up to 6 weeks) [18].

The GC–MS/O [20,21,22,23,24] method can be used for identifying the compounds released from construction materials, including the odour type and intensity recognition. The adopted methodology allows for the determination of the correlation between the chemical character and the concentration of specific fragrant compounds, owing to the human perception of odour. Each of the perceived compounds is identified based on retention time, reference compounds and spectra database. The sense of smell of the people assessing the signal leaving the analyser fulfils the detector’s role.

Olfactometry, which is a part of sensory analysis, measures the odourant perception thresholds, determines the odour intensity, recognises the type of smell and determines its hedonic tone [25,26,27,28,29,30]. This paper presents analyses concerning direct dynamic olfactometry, which is a method of objective determination of odour concentration in gas samples, where a fragrant gas sample is diluted with odourless gas and presented to the testing persons, who are the detectors.

The GC–MS/O hybrid method helps solve the sensory analysis problem of the odour synergy of a mixture, such as the analysed air sample. There is a valve at the chromatographic column end, where the fragrant gas sample is split into two streams. One of them is mixed with humid air and fed through a thermostat-featured conduit onto the odour assessment port, while the other stream goes to the MS detector. Consequently, a chemical compound can be identified on the chromatograph and simultaneously correlated with an odour stimulus, imaged with an aromagram. The intensity of aromagraphic signals depends on the recording method and may vary depending on the applied system (turning a knob, intensity assessment with voice recordings or pushing buttons on controllers). It is a complex issue in laboratory practice, and odour signals occur before the chromatographic identification peaks, causing their mutual offset [8,22].

Qualitative and quantitative odour assessment can be performed for each identified compound leaving the column. Still, quantitative assessment is relative, and a rank (weight) can be assigned, according to the assumed intensity scale. Odour presence alone is the evidence of its concentration exceeding the threshold triggering odour sensation for the particular compound. The duration of the sensory activity of odour stimuli is further information provided by an olfactometric analysis.

## 2. Materials and Methods

The analyses were carried out using a GCMS-QP2010 gas chromatograph (Shimadzu, Tokyo, Japan) featured with TD20 thermal desorption (Shimadzu, Tokyo, Japan) and connected to an olfactometric port enabling odour detection (Phaser, GL Sciences, the Netherlands). An olfactometric port consists of a glass cone blown through with air previously humidified with water (to protect the mucous membrane in the nose). A computational software or pneumatic system′s control module, which enables automatic setting according to the stream division, and offers a much more convenient and accurate setting of the parameters, can be used for calculating the stream′s flow and determining the split ratio of the gas between the olfactometric port and MS detector. A pneumatic system′s control module was used for the tests presented in the paper. The analyses were carried out on Rxi-5Sil MS capillary chromatographic column (30 m × 0.25 mm × 0.25 µm). A two-position four-way valve was installed downstream on the column, offering two operation modes. Position A was used for operation skipping the olfactometric port, owing to which the entire stream is directed to MS. A part of the stream was fed onto the olfactometric port in position B, while the rest went to MS. The split ratio between MS and OLF depends on the pre-set gas flow rates and pressures. In order to obtain the required stream split values, it was necessary to install two restrictors with the correct lengths and diameters (1.5 m × 0.25 m and 5 m × 0.15 m), which helped achieve the desired pressure values. The MS: OLF stream split ratio amounted to 1:10 in the tests presented in the study.

For such an array configuration, it was assumed that two analyses needed to be performed to obtain the most accurate analytical information for two positions (A and B) of the installed two-position valve. The measurements in position B enabled orienting a larger part of the stream onto the olfactometric port, which results in odour stimuli amplification (higher concentration). Unfortunately, the MS spectrum, which is simultaneously recorded, provides a low-intensity signal. Owing to the MS spectrum in position B and MS spectrum in position A, we gained information about the mutual offset of the spectra for both positions of the valve. Position A skips the olfactometric port directing the entire stream onto the MS detector, providing detailed spectra information on the test sample. Summing up, to obtain the most accurate information, odour signals were collected in position B, while the result from the measurement in position A was used for interpreting the mass spectra. Two samples had to be collected for the analyses. Regardless of the valve position, the measurements were carried out in the splitless mode, which enabled amplification of the odour and mass spectra signals.

**Figure 1:**
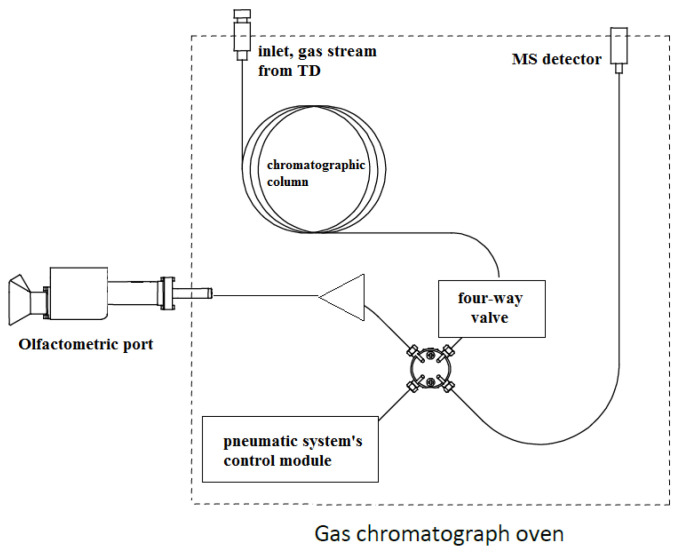
Simplified diagram of the TD–GC–MS/O chromatographic array used for the tests.

**Figure 2:**
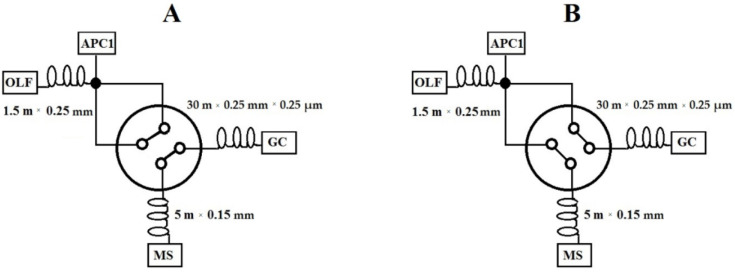
Diagram of a two-position four-way valve in positions (**A**) and (**B**), with the marked chromatographic column and applied restrictors.

Figure 1 shows the system′s configuration used for the tests presented in the paper. Figure 2 shows a diagram of a two-position four-way diagram in positions A and B, with restrictors marked.

The adopted TD–GC–MS/O methodology helps determine the correlation between the chemical compounds and odour signal. This is possible owing to the comparison of the retention times for which the odours were identified with the chemical compounds assigned with the mass spectra available in the NIST 2011 database, assuming the offset between them. All the compounds with the mass spectra matching factors *p* ≥ 80% were regarded as identified. The author described the odour substances according to subjective perception and previous experience [31].

The shifts of the odour signals may result from the pressure differences arising on the GC–MS system, among others, as follows, by: use of a restrictor; the time it takes for the gas stream to travel to the restrictor; the experimenter’s reflexes; passage of the gas stream through the transfer line; human olfactory system responses; information to the brain and finally the decision to record the signal by experimenter.

### 2.1. Air Samples Collected during Renovation and in a Non-Renovated Office Room

The air samples were collected with a dynamic method into tubes filled with Tenax TA© absorber. The samples were simultaneously collected at three measurement points with electronic mass flow controllers from Aparatura Pomiarowa Ochrony Środowiska (local manufacturer). Accredited calibration laboratories regularly calibrate the mass flow controllers. The volume of the collected air samples was 10 L. The sampling rate amounted to 10 L/h. The flow time was measured with an electronic timer.

The chemical compounds captured on the Tenax TA© absorber were desorbed in a thermal desorber in the following conditions: the heated valve’s temperature, 250 °C; feeding line’s temperature, 250 °C; desorption time, five min; helium flow rate, 60 mL/min. After cryogenic focusing, they were released to the carrier gas stream directed to the gas chromatograph.

Chromatographic analysis was performed at the following temperature programme of the GC heater: the initial temperature of 40 °C was maintained for five min and then increased from 10 °C/min to 260 °C; the end temperature amounted to 260 °C and was maintained for one min. The splitless mode was used. The determination limit of the applied method is 1 μg/m^3^.

A slightly chemical odour was present in the renovated room’s air, at the stage of removing the floor staves. The odour originated from the floor layers disturbed during their dismantling, which resulted in increased concentrations of fragrant compounds in the air. The odour had been detectable for the room users before the renovation, but it was less intense.

Air samples from a non-renovated office room used daily were collected for comparison. The room was furnished with plywood racks, desks and chairs. The floor was covered with a fitted carpet. The measurements were carried out in naturally ventilated rooms. All doors and windows had been closed twenty-four hours before the measurement—the rooms were not used or ventilated. The air samples were collected at three representative measurement points located 1.5 m above the floor, away from windows, doors, potential emission sources, or direct sunlight. The temperature in the tested rooms where the samples were collected amounted to 17.7–24.0 °C, and the RH was 29.8–45.2%.

In an office room, a tar adhesive under the parquet flooring was detected. Tar adhesives are purified fractions of raw coal tars and mixtures of raw coal tar or tar oil with coal pitch, having specific physicochemical properties, useful in practical applications. They were used for waterproofing ceilings of buildings as well as for gluing floor slats to the concrete substrate. They included the following: aliphatic, aromatic and heteroaromatic hydrocarbons and Phenol derivatives. Tar adhesives show the highest vapor emissions of Naphthalene, methylnaphthalenes, ethylnaphthalenes, Acenaphthalene, biphenyls, Dibenzofuran, Fluorene, Phenanthrene or Anthracene.

### 2.2. Floor Staves Placed in the Emission Chamber

**Figure 3:**
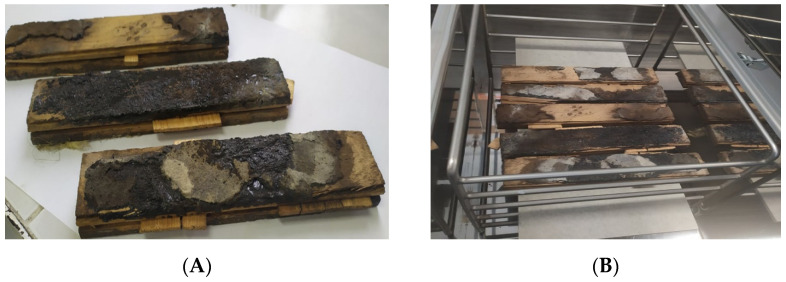
(**A**) Wooden floor staves coated with bitumen, collected from the renovated office room (**B**) The same floor staves placed in the emission chamber.

Several floor staves covered with bitumen-based (Figure 3A) products were collected from the renovated room where the air was sampled for the tests. Then, the staves were placed in a stainless emission chamber with 100 L volume. Finally, the test samples were laid on an inert material—fibre cement panels (Figure 3B). The dimensions of the tested material were suited to the test chamber size and the loading factor, which amounts to L = 0.4 for flooring products. The loading factor is the ratio of the tested material’s area in the reference room to the reference room’s volume (m^2^/m^3^). The overall dimensions of the test sample amounted to 20 cm × 20 cm. The values of the chamber loading factors, reference room’s volume, acceptable size of the test chamber and other test parameters are described in PN-EN 16516 + A1:2020 [32]. The standard assumes air collection from an empty chamber (background) 7 and 28 days after placing the material in the chamber at the specified air flow rate. The absence of the airflow through the chamber to accumulate the compounds released from the test material differed from the standard [32] assumptions.

Moreover, the air samples were collected from the chamber three days into the seasoning. Five Liters of air were collected from each chamber; the collection rate was 10 L/h for 30 min. Two air samples were collected at the same time. The flow time was measured with an electronic timer. Chromatographic analysis of the air samples collected from the chamber was conducted in the same conditions as the air samples collected in the rooms.

### 2.3. Fragments (Chips) of the Materials Placed in Thermal Desorption Tubes

**Figure 4:**
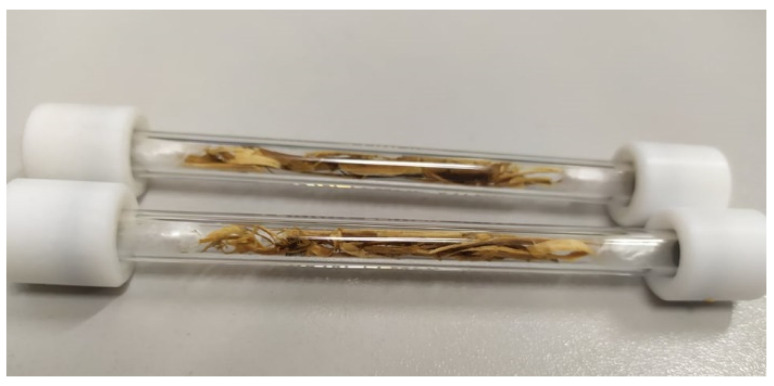
Chips planed from wooden floor staves coated with bituminous products, ready for thermal desorption tests.

Small fragments (chips) were planed from the wooden staves coated with bituminous products and placed in thermal desorption tubes (Figure 4). The weight of the samples was ca. 0.3 g. The samples were subjected to thermal desorption in the following conditions: heated valve’s temperature 250 °C; feeding line’s temperature 250 °C; block’s temperature 70 °C; desorption time 10 min; helium flow rate 60 mL/min. The chromatographic analysis of the collected air samples was performed in the same conditions as the air samples from the rooms and emission chamber.

## 3. Results

### 3.1. Air Samples Collected during the Renovation and in a Non-Renovated Office Room

**Figure 5:**
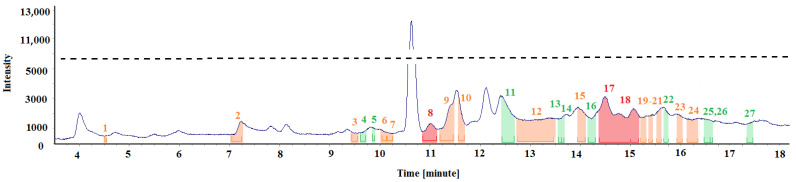
Chromatographic spectrum with the applied odour signals obtained for the air sample collected from the office room during renovation.

Figure 5 and Table 1 show the test results obtained for the air sample collected in a renovated office room. Three air samples were collected, but only one representative spectrum was selected for the description below and carefully analysed. The chromatographic spectra of all the collected samples differed slightly (minor differences in the intensity of individual peaks). However, more significant differences occurred in the odour signals’ identification, which is why the spectrum containing the highest number of identified odours was selected for the analysis presented below.

Figure 5 shows the spectra fragments (marked with different colours) during which specific odours were perceived. The colour type indicates the odour signal’s intensity (strength). The “weak” odours are marked in green, odours with “medium” intensity are orange, and “strong” odours are marked in red. Table 1 summarises the identified odour signals, according to the ordinal number above the peak. The odour description complies with the study author’s subjective perception and previous experiences working with an olfactometric port and the tested matrix.

**Figure 6:**
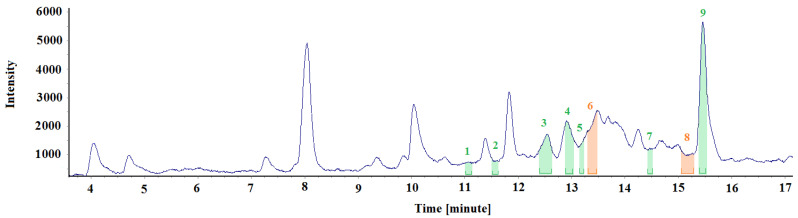
Chromatographic spectrum with the applied odour signals obtained for the air sample collected from the non-renovated office room.

For comparison, Figure 6 and Table 2 show the test results for an air sample collected in a non-renovated office room. Three air samples were also collected in the room, and one representative spectrum was thoroughly analysed, as in the previous case. The chromatographic spectra of all the collected samples were nearly identical (except for the intensity of some peaks). 

Figure 6 shows the spectra fragments, marked with different colours, where specific odours were perceived. The same colour code as described in the previous paragraph was used to identify odour intensities.

### 3.2. Samples of Floor Staves in the Emission Chamber

**Figure 7:**
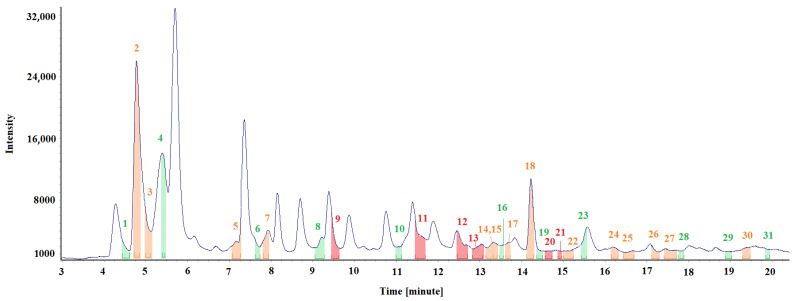
Chromatographic spectrum with the applied odour signals obtained for the air sample collected from the emission chamber after three days.

Figure 7 and Table 3 show the test results obtained for the air sample collected in an emission chamber, where the floor staves were placed. Two air samples were collected three days into their seasoning, but only one representative spectrum was selected for a detailed analysis. The chromatographic spectra of both collected samples differed slightly (minor differences in the intensity of individual peaks). 

Figure 7 shows the spectra fragments (marked with different colours) during which specific odours were perceived. The colour type indicates the odour signal’s intensity (strength). The “weak” odours are marked in green, odours with “medium” intensity are orange, and “strong” odours are marked in red. Table 3 summarises the identified odour signals according to the ordinal number above the peak.

### 3.3. Fragments (Chips) of the Materials Placed in Thermal Desorption Tubes

**Figure 8:**
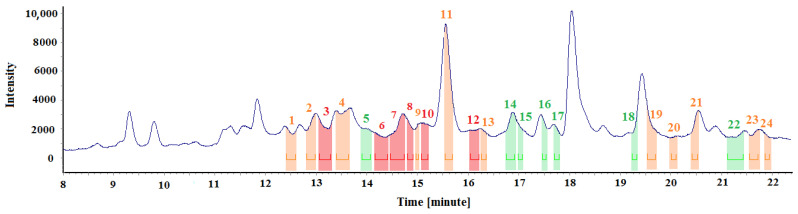
Chromatographic spectrum with the applied odour signals obtained for the samples of floor staves exposed to elevated desorption temperature.

In order to obtain extra analytical information about the tested array, Figure 8 and Table 4 show the test results obtained for the wooden floor staves’ fragments (chips), exposed to emissions at an elevated desorption temperature of 70 °C (Section 2). 

Figure 8 contains the spectra fragments (marked with different colours) during which specific odours were perceived. The same colour code as previously described was used to identify odour intensities. Table 4 summarises the exact durations of the odour signals and the chemical compounds responsible for them, assigned based on spectral data (where applicable).

## 4. Discussion

### 4.1. Air Samples Collected during Renovation and in a Non-Renovated Room

A slight chemical odour was perceived in the renovated room’s air, at the stage of the floor staves’ removal. The odour originated from the floor layers disturbed during dismantling and resulted in a higher airborne concentration of fragrant compounds from bituminous products and wood. An onerous odour, described by the room users even before the renovation, was less intense.

Comparing chromatographs and odour signals from the air samples collected in the non-renovated and renovated room of the same intended use, reveals that the odour signals diversity was higher in the latter ones, and there were more medium and strong intensity signals. Three strong signals, fourteen medium signals and ten weak signals were identified in the renovated room. In the non-renovated room, despite seven weak-intensity signals, two odours were identified to which medium intensity was assigned. Moreover, the compounds identified in the renovated room are present in more significant amounts, confirmed by the intensity scale values.

Terpene hydrocarbons (mostly pleasant odours), such as α-Pinene, Camphene, β-Pinene, 3-Carene, Acetophenone, Linalool, Cis-Verbenol and Pinocarvone were assigned to the odour signals identified in the samples from the renovated room. They are natural compounds of many essential oils, originating from evergreen trees (carenes, pinenes). Moreover, unpleasant odour signals were also identified. They are associated with the smell of naphthalene, tar and old wood. Naphthalene and 1-Methylnaphthalene were assigned to them in the spectrum (Figure 5).

The compounds mentioned above, present in the renovated room’s air, could have originated from the floor materials, i.e., wooden floor staves and insulation and moisture barriers, such as tar paper and adhesive. The released quantities of the compounds were higher because their structures were disturbed.

3-Carene was also identified in the non-renovated room, although in lower concentrations. Moreover, odours were identified coming from glycol ether derivatives, used in industry as solvents for paints, varnishes (released from varnish coats applied to wooden surfaces), dyes and adhesive agents, and components of cleaning agents.

### 4.2. Samples of Floor Staves in the Emission Chamber

The floor panel samples (Figure 3) emitted a strong unpleasant odour, characteristic of bituminous materials. The airflow through the chamber was switched off to accumulate the compounds emitted from the floor materials because the test assumption was to determine the qualitative, rather than quantitative, characteristics of the emitted compounds. As expected, the intensity values of the chromatographic peaks were higher than the values obtained for the air samples collected in the room.

A representative chromatograph was selected for a detailed analysis. An olfactometric analysis helped identify six strong-intensity signals, fourteen medium-intensity signals and eleven weak-intensity signals. However, assigning the likely compounds was impossible for many odour signals, so they were marked as n/a. In such cases, the experimenter’s sense of smell was more sensitive than the sensitivity threshold of the testing apparatus or the non-identified odour signals resulting from the odour synergy or the “echo” of the preceding signals.

The odour signals identified in the air samples collected from the emission chamber, where the floor staves from the renovated room were placed, were mainly assigned to simple aromatic cyclic compounds, such as xylene, phenol, and naphthalene methyl derivatives. Bicyclic aromatic hydrocarbons are characteristic of tar products from coal processing (pitch, coal tar, adhesive, oil from coal tar distillation) and are evidence of the tar adhesive’s presence in the test samples. Tricyclic aromatic hydrocarbons have a high molecular weight and are non-volatile at room temperature; hence, they were not identified in the study.

### 4.3. Fragments (Chips) of the Materials Placed in Thermal Desorption Tubes

The results presented in Figure 8 and Table 4 suggest that wood chip tests contain many odour signals, and their intensity is highly diversified. An olfactometric analysis helped identify six strong signals, eleven medium-intensity signals and seven weak signals. An unpleasant odour of naphthalene characterised most of the signals to which chemical compounds were assigned. The odours were described as chemical, naphthalene, old wood, unpleasant, etc. Since the samples were exposed to a higher temperature than the room temperature during the desorption, heavier and more branched derivatives of aromatic compounds were released from them, including 2,3-Dimethylphenol, 2-Ethyl-5-methylphenol, 4-Ethyl-2-methoxyphenol, 2,6-Dimethyl naphthalene and dibenzofuran.

Desorption was performed at 70 °C. Observations, previous tests and experiments [31] revealed that heating the samples to higher temperatures causes their thermal destruction, manifested by a burnt material odour released from the olfactometric port. Moreover, interpreting the signals present then in the chromatographs is impossible, because either the signals originate from the samples’ thermal degradation products or there are too many signals, especially in the high spectral range.

## 5. Conclusions

Based on the measurements, the conclusion was drawn that the method of collecting the samples and their preparation for the tests is the key aspect of the experiment and must be highly repeatable. Table 1, Table 2, Table 3 and Table 4 and Figure 5, Figure 6, Figure 7 and Figure 8 describe all the odours that were identified during the measurements presented in the article. Apparently, the rest of the compounds present in the test samples were odourless or the odour detection threshold of these compounds was higher than their concentrations in the test samples. Each compound has its own detection threshold; hence, it can be concluded that a high GC–MS signal does not mean a strong odour intensity and vice versa. A low GC–MS signal may correspond to a very intense odour.

The experiment series led to the conclusion that, in order to correlate the odour leaving the olfactometric port with a clear chromatographic signal, two samples, collected simultaneously at two positions of the high-temperature two-position four-way valve, should be analysed. This results from the fact that for valve position B (Section 2), most of the tested gas stream is subjected to sensory analysis. At such an apparatus setting, one-tenth of the stream reaches the MS detector, which results in the spectrum’s weak signal. In such cases, we get a strong odour and insufficient information in the chromatographic spectrum (weak intensity). Therefore, to correlate numerous odour signals with eluting compounds, additional analysis should be carried out on a sample collected simultaneously at the other valve position (position A). A spectrum obtained in this way is applied to the odour signals from the olfactometric port, and hence, the complete information presented in this paper is provided [12,13,14].

In some situations, the human nose is more sensitive than chromatographic detection, which is why an odour stimulus cannot be assigned to any of the eluting compounds.

The quality of the analytes’ chromatographic splitting, meaning the GC–MS analysis conditions, matters for the qualitative assessment of odour. A human is a proper detector in the described method. That is why the factors affecting the assessment have to be stable, i.e., laboratory free of odours, stable temperature and pressure, sequence of the analysed samples, their repeatability and scale used for the odour intensity assessment.

Based on the presented and previous studies [31], the author demonstrated that such compounds as naphthalene, methyl naphthalenes, dimethyl naphthalenes, biphenyl and acenaphthene could be identified in the air, in rooms where tar or asphalt binder was present, and in rooms where the wood was impregnated with chloronaphthalene-based agents [31]. This article [31] provides quantitative and qualitative results, and explains more about the differences between emissions from wooden structures, which were impregnated with tar compositions (creosote oil and Xylamite oil containing tar products), and buildings in which bituminous seal containing hydrocarbon solvents was used. The enumerated compounds seem responsible for the naphthalene-like odour of the air in the rooms.

The fact that some odours are repeated in the different spectra and other odours are not, is related to (1) different concentrations of compounds in the tested samples, (2) various odour detection thresholds and (3) the type of selected detection method (from indoor air, emission chamber, thermal desorption).

In indoor air research, fewer compounds can be identified, compared to materials research. Despite the fact that the odour is often clearly felt in the tested rooms, analytical results often do not show high concentrations of components derived from tar adhesives. The repeatability for air samples taken in one room, at the same time, is practically identical, whereas for samples taken from different rooms, the results are different.

Analyses performed on samples placed in emission chambers or subjected to thermal desorption provide more information on the detectable odours originating from this type of material. The emissions from tar adhesives show a greater concentration and variety of compounds because they are isolated in the test chambers (chamber background subtraction was also used). Besides, the research concerned the floor layers disturbed during their dismantling. The repeatability for the air samples taken from test chambers for the same products is practically identical, whereas, for different tar adhesive products, the emissions differ from each other. This is related to complex tar adhesive compositions.

The fragments (chips) of the materials were subjected to desorption temperatures in which organic compounds are more concentrated. The reproducibility of these chip test results from one sample is very high, but the results are not identical. One should remember that wood chip samples are subjected to thermal desorption at a temperature higher than the temperature in office rooms, so the results supplement the results obtained for air samples tests and tests in emission chambers.

## Figures and Tables

**Table 1:** Identification of odour signals obtained for the air sample collected in an office room during renovation.

No.	Signal Beginning	Signal End	Intensity	Odour Description	Assigned Compound
1	4514	4541	Medium	n/a	n/a
2	7047	7227	Medium	solvent, chemical	m-Xylene
3	9434	9567	Medium	fat, oil, resin	Alpha-Pinene
4	9601	9721	Weak	tar, wood	Camphene
5	9851	9894	Weak	aa	n/a
6	10,017	10,114	Medium	fat, tallow	n/a
7	10,121	10,231	Medium	pine, resin	Beta-pinene
8	10,864	11,141	Strong	pine, resin, forest	3-Carene
9	11,191	11,470	Medium	unpleasant	n/a
10	11,564	11,674	Medium	aa	n/a
11	12,434	12,684	Weak	solvent	Acetophenone
12	12,750	13,500	Medium	spice, orange, citrus	Linalool
13	13,517	13,524	Weak	n/a	n/a
14	13,564	13,670	Weak	solvent	Cis-Verbenol
15	13,970	14,097	Medium	n/a	n/a
16	14,154	14,294	Weak	medicinal	Pinocarvone
17	14,370	14,934	Strong	naphthalene	Naphthalene
18	14,944	15,160	Strong	medicinal, chemical	D-verbenone
19	15,200	15,290	Medium	aa	n/a
20	15,364	15,427	Medium	aa	n/a
21	15,540	15,610	Medium	aa	n/a
22	15,674	15,720	Weak	aa	n/a
23	15,940	16,010	Medium	aa	n/a
24	16,120	16,334	Medium	chemical, tar, mothball	1-Methylnafthalene
25	16,480	16,577	Weak	n/a	n/a
26	16,587	16,620	Weak	n/a	n/a
27	17,317	17,427	Weak	n/a	n/a

aa—as above; n/a—not applicable.

**Table 2:** Identification of the signals obtained for the air sample collected from the non-renovated office room.

No.	Odour Beginning	Odour End	Intensity	Odour Description	Assigned Compound
**1**	11,024	11,117	Weak	chemical	n/a
**2**	11,514	11,584	Weak	citrus	3-Carene
**3**	12,411	12,611	Weak	chemical	2-Ethyl-1-hexanol
**4**	12,897	13,024	Weak	a/a	n/a
**5**	13,154	13,214	Weak	naphthalene	n/a
**6**	13,304	13,444	Medium	plastic	2-Butoxyethyl acetate
**7**	14,444	14,507	Weak	plasticine	n/a
**8**	15,060	15,267	Medium	chemical	Benzoic acid
**9**	15,404	15,517	Weak	plastic	1-(2-butoxyethoxy)ethanol

aa—as above: n/a—not applicable.

**Table 3:** Identification of the signals obtained for the air sample collected from the emission chamber.

No.	Odour Beginning	Odour End	Intensity	Odour Description	Assigned Compound
1	4471	4578	Weak	fat, tallow	n/a
2	4731	4841	Medium	acetic	Acetic acid
3	5001	5114	Medium	n/a	n/a
4	5381	5448	Weak	butter	n/a
5	7094	7238	Medium	plastic	Methylcyclohexane
6	7638	7718	Weak	butter, camphor	Methyl Isobutyl Ketone
7	7808	7928	Medium	a/a	a/a
8	9084	9231	Weak	plastic	m-Xylene
9	9468	9611	Strong	unpleasant	n/a
10	11,008	11,108	Weak	mushroom, musty	Heptanoic acid
11	11,464	11,671	Strong	citrus, fruit, fresh	3-Carene
12	12,468	12,704	Strong	tar, naphthalene	Phenol
13	12,874	13,091	Strong	a/a	m-Cresol
14	13,144	13,324	Medium	a/a	n/a
15	13,331	13,451	Medium	plastic	n/a
16	13,491	13,568	Weak	alcohol, solvent	n/a
17	13,571	13,708	Medium	wood, musty	Acetophenone
18	14,174	14,328	Medium	wood	n/a
19	14,368	14,511	Weak	plastic	n/a
20	14,534	14,724	Strong	naphthalene	Nafthalene
21	14,864	14,988	Strong	naphthalene	Nafthalene
22	14,994	15,234	Medium	tar, wood	n/a
23	15,441	15,541	Weak	n/a	n/a
24	16,161	16,291	Medium	tar, wood	1-Methylnafthalene
25	16,471	16,667	Medium	pleasant	n/a
26	17,121	17,287	Medium	n/a	n/a
27	17,414	17,744	Medium	tar, wood	2-Methylnaphthalene
28	17,757	17,874	Weak	forest fruit	n/a
29	18,891	18,997	Weak	mold, musty	n/a
30	19,321	19,454	Medium	pleasant	n/a
31	19,864	19,911	Weak	plasticine	n/a

aa—as above; n/a—not applicable.

**Table 4:** Identification of the signals obtained for the samples of floor staves exposed to elevated desorption temperature.

No.	Odour Beginning	Odour End	Intensity	Odour Description	Assigned Compound
1	12,421	12,587	Medium	chemical, medicine	Phenol
2	12,817	12,981	Medium	unpleasant	n/a
3	13,064	13,281	Strong	unpleasant	m-Cresol
4	13,397	13,630	Medium	unpleasant	n/a
5	13,904	14,067	Weak	n/a	n/a
6	14,164	14,407	Strong	chemical	o-Cresol
7	14,464	14,740	Strong	unpleasant	n/a
8	14,810	14,917	Strong	unpleasant	2,3-Dimethylphenol
9	14,967	15,017	Medium	pleasant, sweet	n/a
10	15,080	15,207	Strong	naphthalene	Naphthalene
11	15,550	15,684	Medium	n/a	n/a
12	16,054	16,207	Strong	wood	n/a
13	16,257	16,350	Medium	wood	2-Ethyl-5-methylphenol
14	16,757	16,917	Weak	plant, herbal	n/a
15	16,990	17,064	Weak	unpleasant	4-Ethyl-2-methoxyphenol
16	17,464	17,540	Weak	n/a	n/a
17	17,697	17,797	Weak	unpleasant	1-Methylnaphthalene
18	19,237	19,330	Weak	n/a	n/a
19	19,537	19,694	Medium	wood	2,6-Dimethylnaphthalene
20	20,004	20,104	Medium	n/a	n/a
21	20,420	20,527	Medium	n/a	n/a
22	21,120	21,427	Weak	n/a	n/a
23	21,560	21,724	Medium	naphthalene	Dibenzofuran
24	21,857	21,940	Medium	n/a	n/a

aa—as above; n/a—not applicable.
